# Race, Income, and Survival in Stage III Colon Cancer: CALGB 89803 (Alliance)

**DOI:** 10.1093/jncics/pkab034

**Published:** 2021-04-12

**Authors:** Seohyuk Lee, Sui Zhang, Chao Ma, Fang-Shu Ou, Eric G Wolfe, Shuji Ogino, Donna Niedzwiecki, Leonard B Saltz, Robert J Mayer, Rex B Mowat, Renaud Whittom, Alexander Hantel, Al Benson, Daniel Atienza, Michael Messino, Hedy Kindler, Alan Venook, Cary P Gross, Melinda L Irwin, Jeffrey A Meyerhardt, Charles S Fuchs

**Affiliations:** 1 Yale School of Medicine, New Haven, CT, USA; 2 Department of Medical Oncology, Dana-Farber/Partners CancerCare, Boston, MA, USA; 3 Alliance Statistics and Data Management Center, Mayo Clinic, Rochester, MN, USA; 4 Department of Oncologic Pathology, Dana-Farber/Partners CancerCare and Harvard Medical School, Boston, MA, USA; 5 Program in MPE Molecular Pathological Epidemiology, Department of Pathology, Brigham and Women’s Hospital and Harvard Medical School, Boston, MA, USA; 6 Department of Epidemiology, Harvard T.H. Chan School of Public Health, Boston, MA, USA; 7 Broad Institute of MIT and Harvard, Cambridge, MA, USA; 8 Department of Biostatistics and Bioinformatics, Duke University, Durham, NC, USA; 9 Memorial Sloan Kettering Cancer Center, New York, NY, USA; 10 Department of Medical Oncology, Dana-Farber/Partners CancerCare, Boston, MA, USA; 11 Toledo Community Hospital Oncology Program, Toledo, OH, USA; 12 Hôpital du Sacré-Coeur de Montréal, Montreal, Canada; 13 Loyola University Stritch School of Medicine, Naperville, IL, USA; 14 Robert H. Lurie Comprehensive Cancer Center, Northwestern University, Chicago, IL, USA; 15 Virginia Oncology Associates, Norfolk, VA, USA; 16 Southeast Clinical Oncology Research Consortium, Mission Hospitals, Asheville, NC, USA; 17 University of Chicago Comprehensive Cancer Center, Chicago, IL, USA; 18 University of California at San Francisco Comprehensive Cancer Center, San Francisco, CA, USA; 19 Yale School of Medicine, Department of Internal Medicine, New Haven, CT, USA; 20 Yale School of Public Health, New Haven, CT, USA; 21 Yale Cancer Center, Smilow Cancer Hospital and Yale School of Medicine, New Haven, CT, USA; 22 Genentech, South San Francisco, CA, USA

## Abstract

**Background:**

Disparities in colon cancer outcomes have been reported across race and socioeconomic status, which may reflect, in part, access to care. We sought to assess the influences of race and median household income (MHI) on outcomes among colon cancer patients with similar access to care.

**Methods:**

We conducted a prospective, observational study of 1206 stage III colon cancer patients enrolled in the CALGB 89803 randomized adjuvant chemotherapy trial. Race was self-reported by 1116 White and 90 Black patients at study enrollment; MHI was determined by matching 973 patients’ home zip codes with publicly available US Census 2000 data. Multivariate analyses were adjusted for baseline sociodemographic, clinical, dietary, and lifestyle factors. All statistical tests were 2-sided.

**Results:**

Over a median follow-up of 7.7 years, the adjusted hazard ratios for Blacks (compared with Whites) were 0.94 (95% confidence interval [CI] = 0.66 to 1.35, *P *=* *.75) for disease-free survival, 0.91 (95% CI = 0.62 to 1.35, *P *=* *.65) for recurrence-free survival, and 1.07 (95% CI = 0.73 to 1.57, *P *=* *.73) for overall survival. Relative to patients in the highest MHI quartile, the adjusted hazard ratios for patients in the lowest quartile were 0.90 (95% CI = 0.67 to 1.19, *P*_trend_ = .18) for disease-free survival, 0.89 (95% CI = 0.66 to 1.22, *P*_trend_ =* *.14) for recurrence-free survival, and 0.87 (95% CI = 0.63 to 1.19, *P*_trend_ = .23) for overall survival.

**Conclusions:**

In this study of patients with similar health-care access, no statistically significant differences in outcomes were found by race or MHI. The substantial gaps in outcomes previously observed by race and MHI may not be rooted in differences in tumor biology but rather in access to quality care.

Colorectal cancer (CRC) remains the third most common cancer and third leading cause of cancer-related deaths in the United States, despite sustained improvements in CRC incidence, survival, and mortality over the past several decades (1). Blacks experience the greatest CRC burden among all racial groups in the United States, with almost 20% and 40% higher incidence and mortality rates, respectively, relative to Whites (1). Beyond being more likely to be detected at a younger age, CRC in Blacks is also typically diagnosed at a more advanced stage, with lower rates of microsatellite instability and with the tumor in a more proximal location, when compared with that in Whites (2-4).

Data from national health surveillance statistics and individual studies indicate that White CRC patients experience more favorable prognoses than Blacks (2,5-10). A variety of biologic and sociodemographic factors have been proposed to contribute to this disparity, including age stratification (9,11-15), comorbidities (16-20), genetic and biologic mediators (21-25), income (2,18,26), tumor grade (27), location (9,28), and staging (29,30). A recent analysis from the Surveillance, Epidemiology, and End Results program found that Black CRC patients had a 32% higher mortality risk than Whites (31). In addition to racial disparities, an assessment of 2019 US cancer statistics observed a widening gap in CRC mortality across socioeconomic status (SES); compared with the most affluent US counties, CRC mortality is now 35% higher in the poorest counties (1).

Variations in the quality of cancer care provided to patients of racial minority and lower SES backgrounds may contribute to inferior outcomes. Black CRC patients have especially been reported to receive lower-quality care than Whites (32-34). Among CRC patients, Simpson et al. (35) found that Blacks are less likely than Whites to receive a specialist consultation or multimodal therapy, leading to a reduced survival rate that was, however, not statistically significant once adjusted for treatment differences.

Given the inequalities experienced by colon cancer patients from underserved populations, we sought to assess the independent influences of race and median household income (MHI) on patient outcomes within a prospective cohort study nested in a randomized clinical trial (RCT) of adjuvant 5-fluorouracil-based therapy for stage III colon cancer. To our knowledge, this is the first investigation of racial and MHI disparities in CRC outcomes embedded in a RCT, which additionally accounts for dietary and lifestyle factors beyond other clinical and sociodemographic variables. Careful and comprehensive documentation during the trial of patient performance status, pathologic stage, postoperative treatment, and dietary and lifestyle habits allowed concurrent effects of patient, disease, and treatment characteristics to be examined.

## Methods

### Study Population

Patients in this prospective cohort study were recruited from the United States and Canada as participants in the National Cancer Institute (NCI)–sponsored Cancer and Leukemia Group B (CALGB; now part of Alliance for Clinical Trials in Oncology) 89803 adjuvant chemotherapy trial for stage III colon cancer (ClinicalTrials.gov identifier: NCT00003835), comparing weekly 5-fluorouracil (5-FU) and leucovorin to weekly 5-FU, leucovorin, and irinotecan. A total of 1264 patients were enrolled between April 1999 and May 2001, after the first 87 patients of which the protocol was amended such that patients were required to complete a self-administered questionnaire examining diet and lifestyle behaviors twice: once midway through chemotherapy (4 months postsurgery; Questionnaire 1) and again 6 months following chemotherapy treatment completion (14 months postsurgery; Questionnaire 2).

Eligibility required patients to have had a complete surgical resection of the primary tumor within 56 days of trial enrollment; regional lymph node, but no distant, metastases; no prior chemotherapy or radiation treatment for the tumor; a baseline Eastern Cooperative Oncology Group (ECOG) performance status between 0 and 2; and sufficient bone marrow, hepatic, and renal functions. Supplementary Figure 1 (available online) describes the derivation of the final sample sizes of 1206 and 973 patients included in this study for race and MHI analyses, respectively.

CALGB 89803 had long-term follow-up for disease recurrence and overall survival (OS), which continued for 5 and 7 years posttreatment, respectively. Follow-up examinations for disease recurrence occurred annually. OS was assessed via phone calls to patients and their family members as well as information from patient charts, with follow-up occurring every 3 months for the first 2 years, every 4 months for the next 2 years, and yearly for the last 3 years. The National Death Index was used only in cases of loss to follow-up. The last patient in for the trial was in April 2001, and the study was terminated in April 2009 with the long-term follow-up duration having been met.

### Assessment of Patient Race, Insurance Status, and Median Household Income

The race and insurance status of each of the participating patients were self-reported at the time of enrollment as, respectively, Black, White, Hispanic or Latino or Spanish origin, Asian, Native Hawaiian, Native American, Indian, Filipino, or other, and private, Medicare or Medicaid or military, or self-pay or none or unknown. Analyses by race were limited to the 1206 patients who were eligible for CALGB 89803 as described above and whose races were specified as either Black or White. Patients who reported a race other than Black or White were excluded due to very limited power in the other racial categories. MHI was determined by matching the zip codes of patient home addresses, self-reported at the time of enrollment, with publicly available US Census 2000 information. Analyses by MHI were limited to the 973 patients who were eligible for CALGB 89803 as described above and whose MHI data were able to be matched with US census information.

### Dietary Assessment

Patients completed a validated food frequency questionnaire querying consumption of 131 items over the past 3 months, as previously described (36-38). Classification of patients between prudent and Western dietary patterns (39), characterized by high intakes of fruits and vegetables, poultry, and fish vs high intakes of meat, fat, refined grains, and dessert, respectively, was performed following techniques previously described (40). Body mass index, levels of engagement in physical activity, and consistent aspirin use—defined as any aspirin use reported both during (Questionnaire 1) and after completion of adjuvant chemotherapy (Questionnaire 2)—were also recorded.

### Endpoints

The primary endpoint for this study was disease-free survival (DFS), defined as time from study enrollment to tumor recurrence, occurrence of a new primary colon cancer, or death consequent of any cause. Recurrence-free survival (RFS) was defined as time from study enrollment to tumor recurrence, occurrence of a new primary colon tumor, or death with evidence of recurrence, censoring patients who died with no known tumor recurrence at the last documented evaluation. OS was defined as the time from study enrollment to death because of any cause.

### Statistical Analysis

Findings from the CALGB 89803 trial for stage III colon cancer have previously been described (41). As the 2 chemotherapy treatment arms demonstrated similar results, patient data were combined from both treatment arms and analyzed for this study according to categories of race or MHI quartiles. Baseline characteristics were compared between Whites and Blacks and between patients from different income quartiles using Wilcoxon test for continuous variables (age, MHI) and χ^2^ or Fisher exact test for the remaining categorical variables.

The Kaplan-Meier method was performed to estimate the distributions of survival times according to race or MHI. Cox proportional hazards regression was used to determine the associations between race (Black vs White) or MHI (quartiles) and survival outcomes, controlling for potential confounders. The proportional hazards assumptions were graphically assessed and met. Two models were built to incrementally examine the association between race or MHI and the study endpoints. Model 1 was adjusted for age, and model 2 was adjusted for age, sex, treatment arm, T-stage, number of positive lymph nodes, ECOG performance status, tumor location, presence of clinical bowel obstruction or perforation, insurance status, consistent aspirin intake, energy intake, body mass index, physical activity, Western dietary pattern, and prudent dietary pattern, where the last 5 variables were treated as time-varying covariates. We conducted linear trend tests across quartiles of MHI by modeling MHI as a continuous variable and assigning each patient the median value for her or his corresponding quartile. Tests of interaction between race or MHI and potential confounders were assessed by entering the cross-product of race or MHI and the covariate of interest. All statistical tests were 2-sided, and *P* values equal to or less than .05 were considered statistically significant. All analyses were conducted using SAS software (version 9.4; SAS Institute, Cary, NC).

Patient registration and clinical data collection were managed and their analyses performed by the Alliance Statistics and Data Center. The statistical analyses were based on the study database frozen on November 9, 2009. Data quality was ensured by review of data by the Alliance Statistics and Data Center and by the study chairperson following Alliance policies.

All patients signed study-specific informed consent, which was approved by the NCI Cancer Treatment Evaluation Program and each participating site’s institutional review board.

## Results

### Baseline Characteristics According to Race

Within our cohort, 92.5% self-identified as White and the remaining 7.5% as Black. Table 1 summarizes baseline clinical and sociodemographic characteristics of the study cohort according to race. Relative to Whites, Blacks were more likely to have a lower MHI, be female, have a proximal tumor, demonstrate a worse ECOG status, engage in less physical activity, and have a higher Western and a lower prudent dietary pattern.

**Table 1.** Baseline characteristics of 1206 stage III colon cancer patients by race^a^

Characteristic	Race			Total (N = 1206)
	White (n = 1116)	Black (n = 90)	*P* ^b^	
Median age (Q1-Q3), y	61.0 (52.0-69.0)	58.0 (49.0-70.0)	.19	61.0 (52.0-69.0)
Household income, median (Q1-Q3), $	41 256.5 (35 079.0-52 561.0)	32 338.0 (26 757.5-38 878.0)	<.001	40 665.5 (33 967.0-51 668.0)
Sex, No. (%)			.04	
Male	634 (56.8)	41 (45.6)		675 (56.0)
Female	482 (43.2)	49 (54.4)		531 (44.0)
Treatment arm, No. (%)			.59	
5-FU/LV	550 (49.3)	47 (52.2)		597 (49.5)
IFL	566 (50.7)	43 (47.8)		609 (50.5)
T-stage, No. (%)^c^			.19	
T1-2	146 (13.3)	7 (8.3)		153 (13.0)
T3-4	948 (86.7)	77 (91.7)		1025 (87.0)
Missing	22	6		28
Number of positive nodes, No. (%)			.39	
1-3	699 (63.5)	58 (68.2)		757 (63.9)
≥4	401 (36.5)	27 (31.8)		428 (36.1)
Missing	16	5		21
Performance status, No. (%)^d^			.002	
ECOG 0	829 (75.5)	51 (60.0)		880 (74.4)
ECOG 1, 2	269 (24.5)	34 (40.0)		303 (25.6)
Missing	18	5		23
Clinical bowel obstruction or perforation, No. (%)			.57	
No	836 (74.9)	65 (72.2)		901 (74.7)
Yes	280 (25.1)	25 (27.8)		305 (25.3)
Tumor location, No. (%)			.02	
Distal	471 (42.9)	25 (29.8)		496 (42.0)
Proximal	626 (57.1)	59 (70.2)		685 (58.0)
Missing	19	6		25
Insurance status, No. (%)			.75	
Private/self-pay	713 (63.9)	56 (62.2)		769 (63.8)
Medicare/Medicaid/military/other/none	403 (36.1)	34 (37.8)		437 (36.2)
Energy intake in FFQ1, No. (%)			.21	
Median (Q1-Q3)	1970 (1538-2397)	1604 (1272-2325)		
<Median	464 (49.4)	39 (57.4)		503 (50.0)
≥Median	475 (50.6)	29 (42.6)		504 (50.0)
BMI in FFQ1, No. (%)			.21	
Median (Q1-Q3)	27 (24-31)	28 (25-33)		
<Median	474 (50.5)	29 (42.6)		503 (50.0)
≥Median	465 (49.5)	39 (57.4)		504 (50.0)
Physical activity in FFQ1, No. (%)			.003	
Median (Q1-Q3)	5.2 (1.4-15.6)	2.0 (0.25-6.5)		
<Median	457 (48.7)	46 (67.6)		503 (50.0)
≥Median	482 (51.3)	22 (32.4)		504 (50.0)
Western dietary pattern in FFQ1, No. (%)			.04	
Median (Q1-Q3)	−0.13 (−0.60 to 0.53)	−0.33 (−0.99 to 0.08)		
<Median	461 (49.1)	42 (61.8)		503 (50.0)
≥Median	478 (50.9)	26 (38.2)		504 (50.0)
Prudent dietary pattern in FFQ1, No. (%)			.02	
Median (Q1-Q3)	−0.18 (−0.59 to 0.40)	−0.45 (−0.79 to 0.41)		
<Median	460 (49.0)	43 (63.2)		503 (50.0)
≥Median	479 (51.0)	25 (36.8)		504 (50.0)
Consistent aspirin use (FFQ1 and 2), No. (%)			.16	
No	859 (91.5)	66 (97.1)		925 (91.9)
Yes	80 (8.5)	2 (2.9)		82 (8.1)
Reason off study, No. (%)			.03	
Completed planned therapy	833 (74.6)	66 (73.3)		899 (74.5)
Recurrence or death	55 (4.9)	3 (3.3)		58 (4.8)
Adverse events	76 (6.8)	1 (1.1)		77 (6.4)
Others	152 (13.6)	20 (22.2)		172 (14.3)

a Missing value manipulation in following analysis: missing % is less than 5%, and there is a majority category (%>60%), the missing values were recoded into the majority category (T-stage, number of positive nodes, performance status); no majority category (location, proximal or distal), the missing values were recoded as a separate indicator when using as covariates. 5-FU = 5-fluorouracil; BMI = body mass index; ECOG = Eastern Cooperative Oncology Group; FFQ = food frequency questionnaire; IFL = irinotecan, 5-fluorouracil, leucovorin; LV = leucovorin; Q = quartile.

b *P* value based on Wilcoxon test for continuous variables (median household income and age); or χ^2^ or Fisher exact test for categorical variables without missing category. All tests were 2-sided.

c T1-2 = level of invasion through the bowel wall not beyond the muscle layer; T3-4 = level of invasion through the bowel wall beyond the muscle layer.

d Baseline performance status: performance status 0 = fully active; performance status 1 = restricted in physically strenuous activity but ambulatory and able to carry out light work; performance status 2 = ambulatory and capable of all self-care but unable to carry out any work activities, up and about more than 50% of waking hours.

### Association Between Race and Cancer Recurrence or Mortality

**Figure 1.**
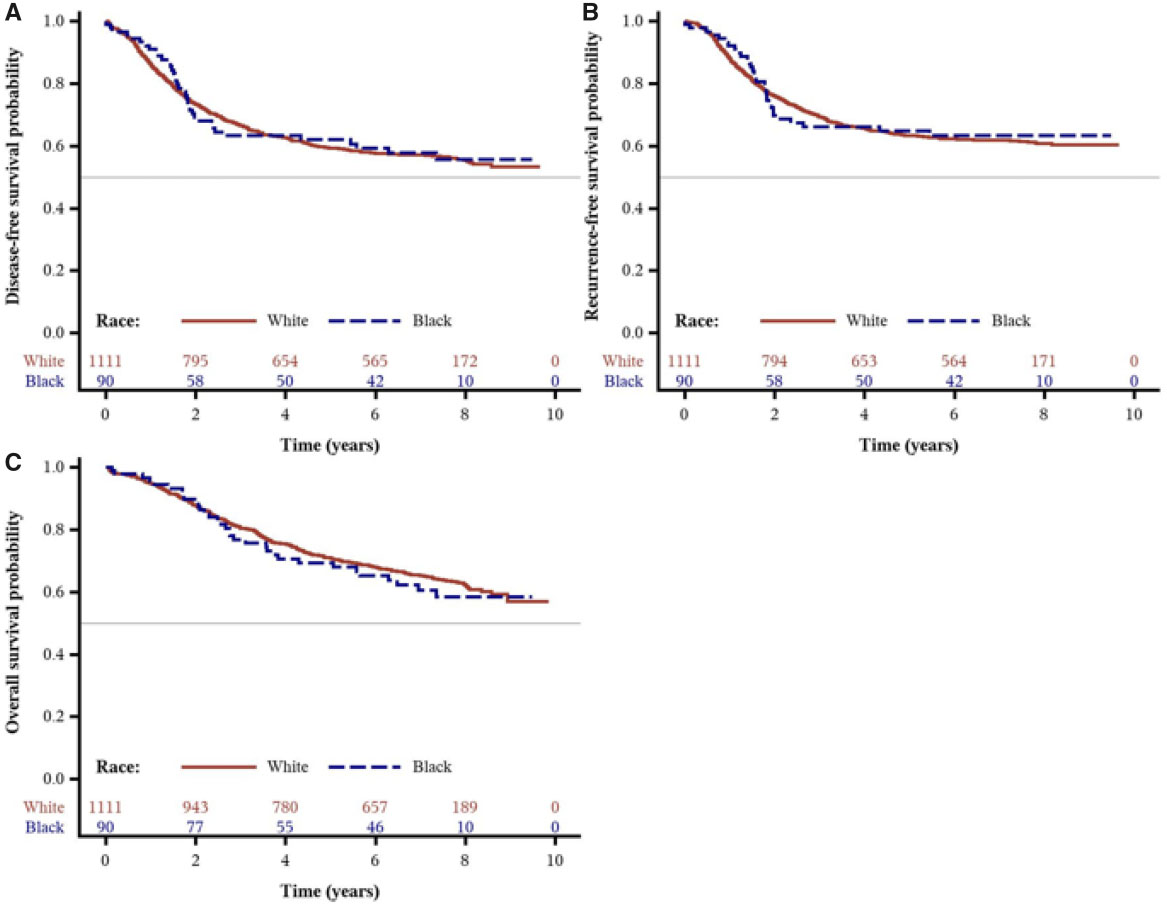
Survival outcomes by race from the Cancer and Leukemia Group B (CALGB) trial 89803. Kaplan-Meier curves of **(A)** disease-free survival, (**B)** recurrence-free survival, and **(C)** overall survival of patients (n = 1206) after a median follow-up of 7.7 years.

Over a median follow-up of 7.7 years, we observed no statistically significant differences in DFS, RFS, or OS between Blacks and Whites in either age-adjusted or multivariable analyses. The distributions of disease-free, recurrence-free, and overall survival times by race are shown in Figure 1. As shown in Table 2 and Supplementary Figure 2 (available online), the adjusted hazard ratios (HRs) for Blacks were 0.94 (95% confidence interval [CI] = 0.66 to 1.35; *P *=* *.75) for DFS, 0.91 (95% CI = 0.62 to 1.35; *P *=* *.65) for RFS, and 1.07 (95% CI = 0.73 to 1.57; *P *=* *.73) for OS, when compared with Whites.

**Table 2.** Race, colon cancer recurrence, and mortality

Outcome	White	Black	*P* ^b^
Disease-free survival			
No. of events/No. at risk	478/1116	37/90	
Age-adjusted only, HR (95% CI)	Referent	1.00 (0.72 to 1.40)	.99
Multivariable adjusted, HR (95% CI)^a^	Referent	0.94 (0.66 to 1.35)	.75
Recurrence-free survival			
No. of events/No. at risk	407/1116	31/90	
Age-adjusted only, HR (95% CI)	Referent	0.96 (0.67 to 1.38)	.82
Multivariable adjusted, HR (95% CI)^a^	Referent	0.91 (0.62 to 1.35)	.65
Overall survival			
No. of events/No. at risk	391/1116	33/90	
Age-adjusted only, HR (95% CI)	Referent	1.16 (0.82 to 1.66)	.40
Multivariable adjusted, HR (95% CI)^a^	Referent	1.07 (0.73 to 1.57)	.73

a Multivariable-adjusted model adjusted for age (continuous), sex (male, female), treatment arm, T-stage (T1-2, T3-4), number of positive nodes (1-3, ≥4), performance status (ECOG 0, ECOG 1-2), tumor location (proximal, distal), clinical bowel obstruction or perforation (yes, no), valid FFQ1 (yes, no), consistent aspirin use (yes, no), insurance status (private/self-pay, Medicare/Medicaid/military/other/none), median household income (quartiles), time-varying energy intake, BMI, physical activity, Western dietary pattern, prudent dietary pattern (all time-varying variables are continuous). BMI = body mass index; CI = confidence interval; ECOG = Eastern Cooperative Oncology Group; FFQ = Food Frequency Questionnaire; HR = hazard ratio.

b *P* values were calculated from the 2-sided Wald test while adjusting for covariates.

### Stratified Analyses of Race by Potential Effect Modifiers

We examined the influence of race on DFS across strata of other potential predictors of patient outcome (Supplementary Table 1, available online). The association between race and patient outcome was not statistically significantly modified across all examined strata of patient, disease, and treatment characteristics. However, in these stratified analyses, statistical power to adequately detect differences was limited by the sample size, and such analyses should be considered exploratory.

### Baseline Characteristics According to Median Household Income

Table 3 represents the baseline patient characteristics of the study cohort according to MHI quartiles. Relative to patients with a higher MHI, those with a lower MHI were more likely to be Black, demonstrate a worse ECOG status, and have a higher Western and a lower prudent dietary pattern, in addition to being less likely to possess private health insurance.

**Table 3.** Baseline characteristics of 973 stage III colon cancer patients by income quartile^a^

Characteristic	Total (n = 973)	Income Quartile				*P* ^b^
		Q1 (n = 243)	Q2 (n = 243)	Q3 (n = 244)	Q4 (n = 243)	
Median household income (Q1-Q3), $	40 542 (33 891-51 894)	30 426 (27 056-32 083)	37 264 (35 604-38 750)	45 087 (42456-48 392)	62 325 (56 876-70 417)	
Median age (Q1-Q3), y	61 (51-69)	62 (52-71)	63 (51-70)	61 (52-69)	59 (50-68)	.07
Race, No. (%)						<.001
White	846 (86.9)	180 (74.1)	214 (88.1)	228 (93.4)	224 (92.2)	
Black	84 (8.6)	48 (19.8)	19 (7.8)	12 (4.9)	5 (2.1)	
Other	43 (4.4)	15 (6.2)	10 (4.1)	4 (1.6)	14 (5.8)	
Sex, No. (%)						.66
Male	534 (54.9)	132 (54.3)	126 (51.9)	139 (57.0)	137 (56.4)	
Female	439 (45.1)	111 (45.7)	117 (48.1)	105 (43.0)	106 (43.6)	
Treatment arm, No. (%)						.51
5-FU/LV	489 (50.3)	127 (52.3)	122 (50.2)	113 (46.3)	127 (52.3)	
IFL	484 (49.7)	116 (47.7)	121 (49.8)	131 (53.7)	116 (47.7)	
T-stage, No. (%)^c^						.65
T1-2	134 (14.0)	39 (16.4)	31 (13.0)	31 (12.8)	33 (13.9)	
T3-4	824 (86.0)	199 (83.6)	208 (87.0)	212 (87.2)	205 (86.1)	
Missing	15	5	4	1	5	
Number of positive nodes, No. (%)						.56
1-3	618 (64.0)	151 (62.7)	162 (67.5)	157 (64.3)	148 (61.7)	
≥4	347 (36.0)	90 (37.3)	78 (32.5)	87 (35.7)	92 (38.3)	
Missing	8	2	3		3	
Performance status, No. (%)^d^						.03
ECOG 0	703 (72.9)	165 (68.8)	179 (74.6)	169 (69.3)	190 (79.2)	
ECOG 1,2	261 (27.1)	75 (31.3)	61 (25.4)	75 (30.7)	50 (20.8)	
Missing	9	3	3		3	
Clinical bowel obstruction or perforation, No. (%)						.18
No	733 (75.3)	185 (76.1)	192 (79.0)	172 (70.5)	184 (75.7)	
Yes	240 (24.7)	58 (23.9)	51 (21.0)	72 (29.5)	59 (24.3)	
Tumor location, No. (%)						.95
Distal	411 (42.6)	99 (41.3)	102 (42.5)	105 (43.0)	105 (43.8)	
Proximal	553 (57.4)	141 (58.8)	138 (57.5)	139 (57.0)	135 (56.3)	
Missing	9	3	3		3	
Insurance status, No. (%)						.003
Private/Self-Pay	626 (64.3)	140 (57.6)	146 (60.1)	165 (67.6)	175 (72.0)	
Medicare/Medicaid/military/other/none	347 (35.7)	103 (42.4)	97 (39.9)	79 (32.4)	68 (28.0)	
Energy intake in FFQ1						.22
Median (Q1-Q3)	—	1963 (1517-2418)	1960 (1473-2403)	1804 (1406-2258)	1988 (1548-2391)	
<Median, No. (%)	403 (50.1)	96 (49.0)	95 (49.0)	116 (56.0)	96 (46.2)	
≥Median, No. (%)	402 (49.9)	100 (51.0)	99 (51.0)	91 (44.0)	112 (53.8)	
BMI in FFQ1						.88
Median (Q1-Q3)	—	27 (24-31)	27 (24-32)	28 (24-31)	27 (24-31)	
<Median, No. (%)	402 (49.9)	101 (51.5)	99 (51.0)	99 (47.8)	103 (49.5)	
≥Median, No. (%)	403 (50.1)	95 (48.5)	95 (49.0)	108 (52.2)	105 (50.5)	
Physical activity in FFQ1						.07
Median (Q1-Q3)	—	3.5 (0.87-13)	6.0 (0.8-19)	4.6 (1.2-15)	6.1 (1.3-17)	
<Median, No. (%)	402 (49.9)	112 (57.1)	93 (47.9)	105 (50.7)	92 (44.2)	
≥Median, No. (%)	403 (50.1)	84 (42.9)	101 (52.1)	102 (49.3)	116 (55.8)	
Western dietary pattern in FFQ1						.008
Median (Q1-Q3)	—	−0.1 (−0.64 to 0.85)	0.05 (−0.60 to 0.57)	−0.26 (−0.64 to 0.31)	−0.20 (−0.67 to 0.28)	
<Median, No. (%)	402 (49.9)	89 (45.4)	82 (42.3)	118 (57.0)	113 (54.3)	
≥Median, No. (%)	403 (50.1)	107 (54.6)	112 (57.7)	89 (43.0)	95 (45.7)	
Prudent dietary pattern in FFQ1						<.001
Median (Q1-Q3)	—	−0.36 (−0.71 to 0.29)	−0.23 (−0.64 to 0.37)	−0.29 (−0.67 to 0.40)	−0.07 (−0.51 to 0.41)	
<Median, No. (%)	402 (49.9)	110 (56.1)	100 (51.5)	113 (54.6)	79 (38.0)	
≥Median, No. (%)	403 (50.1)	86 (43.9)	94 (48.5)	94 (45.4)	129 (62.0)	
Consistent aspirin use (FFQ1 and 2), No. (%)						.14
No	731 (90.8)	174 (88.8)	178 (91.8)	195 (94.2)	184 (88.5)	
Yes	74 (9.2)	22 (11.2)	16 (8.2)	12 (5.8)	24 (11.5)	
Reason off study, No. (%)						.78
Completed planned therapy	721 (74.1)	182 (74.9)	172 (70.8)	181 (74.2)	186 (76.5)	
Recurrence or death	43 (4.4)	10 (4.1)	10 (4.1)	12 (4.9)	11 (4.5)	
Adverse events	64 (6.6)	13 (5.4)	18 (7.4)	20 (8.2)	13 (5.4)	
Others	145 (14.9)	38 (15.6)	43 (17.7)	31 (12.7)	33 (13.6)	

a Missing value manipulation in following analysis: missing % is less than 5% the missing values were recoded into the majority category (T-stage, number of positive nodes, performance status, tumor location). 5-FU = 5-fluorouracil; BMI = body mass index; FFQ = food frequency questionnaire; IFL = irinotecan, 5-fluorouracil, leucovorin; LV = leucovorin; Q = quartile.

b Two-sided *P* value/corr based on 1) Spearman correlation for age or 2) *P* value from χ^2^ test for categorical variables without missing category.

c T1-2 = level of invasion through the bowel wall not beyond the muscle layer; T3-4 = level of invasion through the bowel wall beyond the muscle layer.

d Baseline performance status: performance status 0 = fully active; performance status 1 = restricted in physically strenuous activity but ambulatory and able to carry out light work; performance status 2 = ambulatory and capable of all self-care but unable to carry out any work activities, up and about more than 50% of waking hours.

### Impact of Income on Cancer Recurrence or Mortality

**Figure 2.**
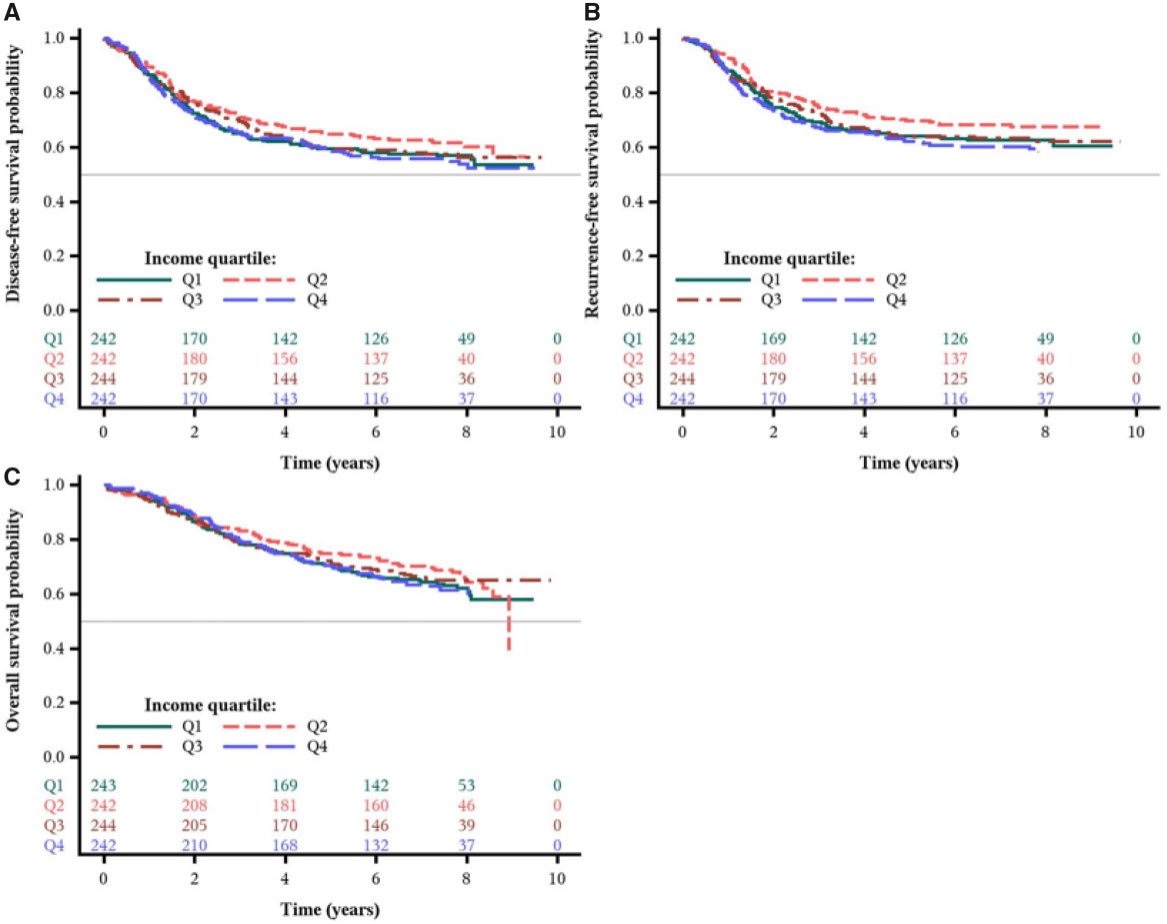
Survival outcomes by income quartile from the Cancer and Leukemia Group B (CALGB) trial 89803. Kaplan-Meier curves of **(A)** disease-free survival, **(B)** recurrence-free survival, and **(C)** overall survival of patients (n = 973) after a median follow-up of 7.7 years.

Over a median follow-up of 7.7 years, we observed no statistically significant differences in patient outcomes across MHI quartiles in either age-adjusted or multivariable analyses. The distributions of disease-free, recurrence-free, and overall survival times by MHI are shown in Figure 2. As shown in Table 4 and Supplementary Figure 3 (available online), the fully adjusted hazard ratios for patients in the lowest quartile of MHI were 0.90 (95% CI = 0.67 to 1.19, *P*_trend_ = .18) for DFS, 0.89 (95% CI = 0.66 to 1.22, *P*_trend_ = .14) for RFS, and 0.87 (95% CI = 0.63 to 1.19, *P*_trend_ = .23) for OS, relative to patients in the highest quartile. Moreover, we examined the independent effect of insurance status (private/self-pay vs Medicare/Medicaid/military/other/none) on patient outcomes and found no statistically significant associations between insurance status and cancer recurrence or mortality (Supplementary Table 2, available online).

**Table 4.** Income quartile, colon cancer recurrence, and mortality

Outcome	Income quartile				*P*_trend_ ^a^
	Q4	Q3	Q2	Q1	
Median household income (Q1-Q3), $	62 325 (56 876-70 417)	45 087 (42 457 to 48 393)	37 264 (35 604 to 38 750)	30 426 (27 056 to 32 083)	
Disease-free survival					
No. of events/No. at risk	107/243	101/244	92/243	103/243	
Age-adjusted only, HR (95% CI)	Referent	0.91 (0.69 to 1.19)	0.80 (0.60 to 1.05)	0.94 (0.71 to 1.23)	.35
Multivariable-adjusted HR (95% CI)^b^	Referent	0.83 (0.63 to 1.10)	0.75 (0.56 to 0.99)	0.90 (0.67 to 1.19)	.18
Recurrence-free survival					
No. of events/No. at risk	94/243	85/244	73/243	86/243	
Age-adjusted only, HR (95% CI)	Referent	0.88 (0.65 to 1.18)	0.73 (0.54 to 0.99)	0.91 (0.68 to 1.22)	.21
Multivariable-adjusted HR (95% CI)^b^	Referent	0.81 (0.60 to 1.09)	0.69 (0.50 to 0.94)	0.89 (0.66 to 1.22)	.14
Overall survival					
No. of events/No. at risk	87/243	80/244	79/243	88/243	
Age-adjusted only, HR (95% CI)	Referent	0.88 (0.65 to 1.20)	0.83 (0.62 to 1.13)	0.97 (0.72 to 1.31)	.59
Multivariable-adjusted HR (95% CI)^b^	Referent	0.79 (0.58 to 1.08)	0.78 (0.57 to 1.06)	0.87 (0.63 to 1.19)	.23

a *P*_trend_ = linear effect with quartile medians. Test was 2-sided. BMI = body mass index; CI = confidence interval; ECOG = Eastern Cooperative Oncology Group; FFQ = Food Frequency Questionnaire; HR = hazard ratio; Q = quartile.

b Multivariable-adjusted model adjusted for age (continuous), sex (male, female), treatment arm, T-stage (T1-2, T3-4), number of positive nodes (1-3, ≥4), performance status (ECOG 0, ECOG 1-2), tumor location (proximal, distal, or missing), clinical bowel obstruction or perforation (yes, no), race (White, Black, other), valid FFQ1 (yes, no), consistent aspirin use (yes, no), time-varying energy intake, BMI, physical activity, Western dietary pattern, prudent dietary pattern (all time-varying variables are continuous), insurance status (private/self-pay, Medicare/Medicaid/military/other/none).

### Stratified Analyses of Median Household Income by Potential Effect Modifiers

We further examined whether the influence of MHI on DFS differed across strata of other potential predictors of patient outcome (Supplementary Table 3, available online). The association between MHI and patient outcome was not statistically significantly modified across most examined strata of patient, disease, and treatment characteristics. We did observe statistically significant interactions between income and number of positive lymph nodes (*P*_interaction_ = .02) and between income and ECOG status (*P*_interaction_ = .02), though these findings were not corrected for multiple hypothesis testing. As previously mentioned, in these stratified analyses, statistical power to adequately detect differences was limited by the sample size, and such analyses should be considered exploratory.

### Joint Impact of Race and MHI on Cancer Recurrence or Mortality

Finally, in exploratory analyses, we examined the joint effect of both race and MHI on patient outcomes (Supplementary Table 4, available online) and found no statistically significant associations. Relative to Whites with a household income above the cohort median, Blacks with a household income below the cohort median did not experience statistically significant differences in DFS, RFS, or OS.

## Discussion

In this prospective cohort of resected stage III colon cancer patients enrolled in a postoperative adjuvant chemotherapy clinical trial, neither race nor MHI was statistically significantly associated with an increased risk of cancer recurrence or mortality. These findings contrast with prior studies, including national surveillance data, which found CRC patients who are Black or from lower SES backgrounds generally experience worse outcomes (1). Our study is, to our knowledge, the first investigation into racial and MHI disparities in colon cancer outcomes embedded in an RCT that additionally accounts for dietary and lifestyle factors beyond other clinical and sociodemographic variables, thereby benefitting from a more robust multivariate analysis than prior studies.

A recent analysis of US cancer statistics observed inequalities in CRC mortality not only by race but also increasingly by SES (1). Cancer mortality-associated SES disparities have worsened in the United States over the past 3 decades. For CRC, mortality rates in the early 1970s for men living in the least affluent counties were 20% lower than for those in affluent ones but are now 35% greater (1). Considered the most prominent trend reversal among all cancer types, this shift in CRC mortality has been cited to be, in part, a consequence of underserved populations experiencing slower receipt of treatment advances (1). Indeed, being both Black and from a lower SES background has even been associated with reduced rates of receiving any cancer treatment (42). In that analysis, physicians treating low-income or predominantly Black patients with colon cancer were found to be 30% and 22% less likely to adhere to guideline-recommended treatments relative to those treating high-income or no Black patients, respectively (42).

Identifying and eliminating barriers to access of care is a critical priority in health care. In the context of the Department of Veterans Affairs hospital system—an equal-access health-care setting—Akerley et al. (43) found no differences in treatment or overall survival between Black and White male veterans. In a multisite NCI-sponsored clinical trial of patients with metastatic CRC, Blacks and Whites receiving standardized treatments experienced similar OS and time-to-progression rates (44). Consistent with our study, other studies have found that CRC outcomes-associated racial disparities between Blacks and Whites are minimal or nonexistent when treatment differences are eliminated (16,18,35,45-47). Notably, Blacks and Whites in our cohort experienced similar outcomes despite Blacks having presented with a worse ECOG performance status and a greater likelihood of having proximal tumors, both of which are generally considered poor prognostic factors.

We directly examined the impact of diet and lifestyle factors and found differences in dietary and lifestyle patterns between Blacks and Whites and between those from lower and higher MHI backgrounds. Such behaviors have long been demonstrated to influence CRC risk and outcomes (39,48-54), and disparities in the prevalence of healthier lifestyle behaviors should be addressed.

Assessing relationships between race or MHI and colon cancer patient outcomes through an RCT offers several strengths. By studying patients enrolled in a clinical trial, we potentially reduced the biases introduced by differences in access to health-care resources unavoidable in population-based cancer registries. Moreover, as all patients in this study met the same enrollment criteria and received adjuvant 5-FU–based chemotherapy, confounding by patient characteristics or the nature of therapy was minimized. Finally, all patients had stage III colon cancer, minimizing the effect of disease stage heterogeneity on outcomes.

Our study is not without limitations. Among our cohort of 1206 patients, only 90 self-identified as Black. Patients who choose to enroll in clinical trials may differ from the general population: they must meet specific eligibility criteria, be chosen as appropriate candidates, and have the motivation to participate. Black patients who ultimately were not offered or had declined participation may be clinically significantly different from those who had participated; nonetheless, the overall outcomes for patients in this trial were comparable with those of a similarly staged population in the Surveillance, Epidemiology, and End Results database. Moreover, CALGB 89803 enrolled patients from both community and academic centers across North America, thereby lowering the likelihood of biased sampling, and the cohort appears to have characteristics representative of the larger population of stage III colon cancer patients. MHI was determined indirectly using zip codes and publicly available US census data as a proxy. While individual, patient-specific income data would provide greater fidelity than zip code–block data, census-block data have identified important disparities in access to care and patient outcomes (55,56). Given the observational nature of our study, we cannot completely exclude the possibility that the statistically nonsignificant associations found between race or MHI and patient outcomes are attributable to confounding variables or residual confounding. However, our findings remained consistent even after controlling for both known and suspected patient outcome predictors.

In conclusion, neither race nor MHI was statistically significantly associated with colon cancer recurrence or mortality in this cohort of stage III patients treated within an RCT. Our findings suggest the substantial gap in outcomes observed between White and Black CRC patients and the growing disparity in outcomes across SES (1) may be rooted in differences in access to and receipt of quality care, rather than in tumor biology. Indeed, Adamson et al. (57) recently found that Medicaid expansion under the Affordable Care Act reduced racial disparities in receiving timely cancer treatment. Our study highlights the need to improve access to quality care for patients across all segments of the population, and especially so for traditionally underserved populations. Efforts by the NCI and US cancer centers to increase enrollment of underrepresented minorities (58,59) into clinical trials (60-62) may help both to ensure the delivery of high-quality care to traditionally underserved populations and to allow for further examination of potential differences in treatment and tumor biology between diverse subgroups.

## Funding

This work was supported by the National Cancer Institute of the National Institutes of Health under award numbers U10CA180821, U10CA180882, and U24CA196171 (to the Alliance for Clinical Trials in Oncology); UG1CA233180, UG1CA233327, UG1CA189858, UG1CA233290, U10CA180867, U10CA138561, U10CA180791, UG1CA233337, R01CA149222, and P30CA016359; U10CA180888 (SWOG); U10CA180820 and UG1CA233320 (ECOG-ACRIN); https://acknowledgments.alliancefound.org. Also supported in part by funds from Pharmacia & Upjohn Company (now Pfizer Oncology; to C.S. Fuchs), the Stand-Up-to-Cancer Colorectal Cancer Dream Team (C.S. Fuchs, grant number: SU2C-AACR-DT22-17), NIH R01 CA169141, NIH R01 CA118553, and NIH P50 CA127003 to C.S. Fuchs, and NIH R35 CA197735 to S. Ogino. Stand Up To Cancer is a division of the Entertainment Industry Foundation. Research grants are administered by the American Association for Cancer Research, the Scientific Partner of SU2C.

## Notes

**Role of the funders:** The sponsors did not participate in the design or conduct of the study; collection, management, analysis, or interpretation of the data; or the preparation, review, or approval of the manuscript.

**Disclosures:** Charles Fuchs reports consulting role for Agios, Amylin Pharmaceuticals, Astra-Zeneca, Bain Capital, CytomX Therapeutics, Daiichi-Sankyo, Eli Lilly, Entrinsic Health, Evolveimmune Therapeutics, Genentech, Merck, Taiho, and Unum Therapeutics. He also serves as a Director for CytomX Therapeutics and owns unexercised stock options for CytomX and Entrinsic Health. He is a co-founder of Evolveimmune Therapeutics and has equity in this private company. He had provided expert testimony for Amylin Pharmaceuticals and Eli Lilly. In March 2021, he became an employee of Genentech. The other authors have no relevant conflicts of interest.

**Author contributions:** Conceptualization: CF; Data Curation: SZ, CM; Formal Analysis: SZ, CM; Funding Acquisition: CF; Investigation: SL, CF; Methodology: CF; Validation: FO, EW; Visualization: SL, SZ, CM; Writing, Original Draft: SL, CF; Writing, Review & Editing: SL, SZ, CM, FO, EW, SO, DN, LS, RJM, RBM, RW, AH, AB, DA, MM, HK, AV, CP, MI, JM, CF.

**Disclaimer:** The content is solely the responsibility of the authors and does not necessarily represent the official views of the National Institutes of Health.

## Data Availability

Data are from the Alliance for Clinical Trials in Oncology. Investigators may request access to this data per Alliance protocol as outlined below and as detailed at https://www.allianceforclinicaltrialsinoncology.org/main/public/standard.xhtml?path=%2FPublic%2FDatasharing. Per NCI National Clinical Trials Network (NCTN) guidelines, any investigator may submit a request for data from published Alliance or legacy ACOSOG, CALGB, or NCCTG trials. To submit a data request, the investigator should complete an Alliance Data Sharing Request Form and send it by e-mail to gro. NTCNecnailla@stpecnoc. Once received, the request will be forwarded to the Alliance Statistics and Data Center (SDC). The SDC will confirm the availability of the data. Once the SDC confirms availability, the investigator will be asked to provide documentation of Institutional Review Board (IRB) approval or exemption from their institution, as well as to submit an Alliance data release agreement. Once the IRB documentation and the data release agreement are received from the requesting investigator, the SDC will be notified that the requested data may be released. Questions about the process may be directed to gro. NTCNecnailla@stpecnoc.

## Supplementary Material

pkab034_Supplementary_Data
