# Supplementary material for: Race, Income, and Survival in Stage III Colon Cancer: CALGB 89803 (Alliance)
Source: JNCI Cancer Spectr. 2021 Apr 12;5(3):pkab034. doi: 10.1093/jncics/pkab034 (PMC8178799; doi:10.1093/jncics/pkab034)

SUPPLEMENTARY MATERIALS

Supplementary Table 1. Disease-free survival by race stratified by clinicopathological and FFQ features.[a]

Subgroup	Count (%)	DFS Events/At-Risk White	DFS Events/At-Risk Black	HR – White	HR – Black	P-value	$P_{interaction}$[b]
Age, years							0.98
>50	943(78.2)	390/880	26/63	Ref	0.85(0.54-1.33)	0.47	
≤50	263(21.8)	88/236	11/27	Ref	0.86(0.44-1.69)	0.65	
Sex							0.22
Male	675(56.0)	286/634	20/41	Ref	1.08(0.65-1.79)	0.77	
Female	531(44.0)	192/482	17/49	Ref	0.68(0.39-1.18)	0.17	
Insurance status							0.97
Private/Self-pay	769 (63.8)	280/713	20/56	Ref	0.86(0.52-1.44)	0.58	
Medicare/Medicaid/military/other/none	437 (36.2)	198/403	17/34	Ref	0.85(0.50-1.46)	0.57	
Treatment arm							0.39
5-FU/LV	597(49.5)	225/550	22/47	Ref	1.00(0.61-1.65)	0.99	
IFL	609(50.5)	253/566	15/43	Ref	0.72(0.41-1.26)	0.26	
T-stage							0.55
T1,2	153(12.7)	35/146	1/7	Ref	0.48(0.06-3.50)	0.47	
T3,4	1053(87.3)	443/970	36/83	Ref	0.88(0.60-1.30)	0.51	
Number of positive nodes							0.80
1-3	778(64.5)	277/715	23/63	Ref	0.83(0.52-1.33)	0.43	
4+	428(35.5)	201/401	14/27	Ref	0.91(0.50-1.67)	0.76	
Performance status [c]							0.98
ECOG 0	903(74.9)	343/847	21/56	Ref	0.86(0.53-1.40)	0.54	
ECOG 1,2	303(25.1)	135/269	16/34	Ref	0.85(0.48-1.52)	0.59	
Clinical bowel perforation or obstruction							0.95
No	901(74.7)	340/836	25/65	Ref	0.86(0.56-1.33)	0.51	
Yes	305(25.3)	138/280	12/25	Ref	0.84(0.41-1.72)	0.63	
Tumor location							0.19
Distal	496(41.0)	186/471	12/25	Ref	1.24(0.65-2.34)	0.51	
Proximal	710(59.0)	292/645	25/65	Ref	0.74(0.47-1.17)	0.20	
BMI in FFQ1							0.81

Subgroup	Count (%)	DFS Events/At-Risk White	DFS Events/At-Risk Black	HR – White	HR – Black	P-value	$P_{interaction}$[b]
<Median	503(50.0)	191/474	10/29	Ref	0.73(0.37-1.45)	0.37	
≥Median	504(50.0)	193/465	15/39	Ref	0.81(0.45-1.49)	0.50	
Physical activity in FFQ1							0.60
<Median	503(50.0)	197/457	16/46	Ref	0.71(0.41-1.26)	0.24	
≥Median	504(50.0)	187/482	9/22	Ref	0.92(0.42-2.00)	0.83	
Western dietary pattern in FFQ1							0.56
<Median	503(50.0)	185/461	16/42	Ref	0.83(0.47-1.48)	0.53	
≥Median	504(50.0)	199/478	9/26	Ref	0.63(0.31-1.31)	0.22	
Prudent dietary pattern in FFQ1							0.77
<Median	503(50.0)	211/460	18/43	Ref	0.79(0.46-1.37)	0.40	
≥Median	504(50.0)	173/479	7/25	Ref	0.68(0.30-1.57)	0.37	

Abbreviations: 5-FU = 5-fluorouracil; LV = leucovorin; IFL = irinotecan, 5-fluorouracil, leucovorin; FFQ = food frequency questionnaire; BMI = body mass index

[a] Multivariable-adjusted model adjusted for age (continuous), sex (male, female), treatment arm, T-stage (T1-2, T3-4), number of positive nodes (1-3, 4+), performance status (ECOG 0, ECOG 1-2), tumor location (proximal, distal), clinical bowel obstruction or perforation (yes, no), valid FFQ1 (yes, no), consistent aspirin use (yes, no), insurance status (private/Self-pay, Medicare/Medicaid/military/other/none), median household income (quartiles), time-varying energy intake, BMI, physical activity, Western dietary pattern, prudent dietary pattern (all time-varying variables are continuous).

[b] Interaction term built as a cross-product of race and the covariate of interest as binary variables.

[c] Baseline performance status: Performance status 0 = fully active; Performance status 1 = restricted in physically strenuous activity but ambulatory and able to carry out light work; Performance status 2 = ambulatory and capable of all self-care but unable to carry out any work activities, up and about more than 50% of waking hour.

Supplementary Table 2. Insurance status, colon cancer recurrence, and mortality.

N=1264	Private/Self-Pay	Medicare/Medicaid/military/other/none	P-value
Household income, Median(Q1-Q3)	41475 (35077-53422)	37969 (32685-47082)	
Disease-free survival			
# Event/At Risk	#312/804	#219/460	
Age-adjusted only, HR (95% CI)	Ref	1.21 (0.99 - 1.49)	0.07
Multivariable adjusted, HR (95% CI) [a]	Ref	1.17 (0.95 - 1.44)	0.14
Recurrence-free survival			
# Event/At Risk	#280/804	#171/460	
Age-adjusted only, HR (95% CI)	Ref	1.17 (0.93 - 1.46)	0.17
Multivariable adjusted, HR (95% CI) [a]	Ref	1.13 (0.90 - 1.42)	0.28
Overall survival			
# Event/At Risk	#248/804	#191/460	
Age-adjusted only, HR (95% CI)	Ref	1.23 (0.98 - 1.54)	0.07
Multivariable adjusted, HR (95% CI) [a]	Ref	1.17 (0.93 - 1.47)	0.17

[a] Multivariable-adjusted model adjusted for age (continuous), sex (male, female), treatment arm, T-stage (T1-2, T3-4), number of positive nodes (1-3, 4+), performance status (ECOG 0, ECOG 1-2), tumor location (proximal, distal, or missing), clinical bowel obstruction or perforation (yes, no), race (White, Black, other), valid FFQ1 (yes, no), consistent aspirin use (yes, no), time-varying energy intake, BMI, physical activity, Western dietary pattern, prudent dietary pattern (all time-varying variables are continuous).

Supplementary Table 3. Disease-free survival by income stratified by clinicopathological and FFQ features.[a,b]

DFS Subgroup	Count (%)	DFS HR – Q4	DFS HR – Q3	DFS HR – Q2	DFS HR – Q1	P-value	$P_{interaction}$[c]
Age, years							0.35
≤60	477(49.0)	Ref	0.71(0.47-1.08)	0.65(0.43-0.99)	0.82(0.54-1.23)	0.15	
>60	496(51.0)	Ref	0.88(0.60-1.29)	1.04(0.71-1.52)	0.94(0.64-1.38)	0.89	
Race							0.76
White	846(91.0)	Ref	0.75(0.56-1.01)	0.81(0.60-1.08)	0.76(0.56-1.02)	0.07	
Black	84(9.0)	Ref	0.90(0.32-2.51)	0.91(0.34-2.47)	0.97(0.36-2.66)	0.98	
Sex							0.55
Male	534(54.9)	Ref	0.74(0.51-1.07)	0.73(0.50-1.04)	0.79(0.55-1.15)	0.12	
Female	439(45.1)	Ref	0.75(0.49-1.15)	0.79(0.52-1.21)	0.95(0.62-1.46)	0.58	
Insurance status							0.45
Private/Self-pay	347(35.7)	Ref	0.80(0.52-1.23)	0.93(0.60-1.42)	0.93(0.60-1.46)	0.69	
Medicare/Medicaid/military/other/none	626(64.3)	Ref	0.76(0.53-1.10)	0.76(0.53-1.09)	0.74(0.51-1.07)	0.09	
Treatment arm							0.55
5FU/LV	489(50.3)	Ref	0.59(0.39-0.89)	0.80(0.54-1.18)	0.92(0.63-1.36)	0.60	
IFL	484(49.7)	Ref	0.90(0.61-1.32)	0.78(0.52-1.16)	0.79(0.53-1.18)	0.16	
T-stage							0.26
T1,2	134(14.0)	Ref	0.40(0.15-1.09)	0.13(0.03-0.60)	0.83(0.36-1.94)	0.15	
T3,4	824(86.0)	Ref	0.86(0.65-1.16)	0.82(0.61-1.10)	0.91(0.68-1.23)	0.36	
Number of positive nodes							0.02
1-3	618(64.0)	Ref	0.83(0.57-1.21)	0.81(0.56-1.18)	1.16(0.81-1.68)	0.81	
4+	347(36.0)	Ref	0.68(0.45-1.03)	0.63(0.42-0.96)	0.57(0.37-0.89)	0.006	
Performance status [d]							0.02
ECOG 0	703(72.9)	Ref	0.76(0.55-1.05)	0.65(0.47-0.92)	0.68(0.48-0.96)	0.009	

DFS Subgroup	Count (%)	DFS HR – Q4	DFS HR – Q3	DFS HR – Q2	DFS HR – Q1	P-value	$P_{interaction}$[c]
ECOG 1,2	261(27.1)	Ref	0.96(0.58-1.59)	0.91(0.55-1.52)	1.48(0.91-2.43)	0.32	
Clinical bowel perforation or obstruction							0.51
No	733(75.3)	Ref	0.83(0.60-1.15)	0.83(0.60-1.16)	0.82(0.59-1.15)	0.20	
Yes	240(24.7)	Ref	0.71(0.42-1.21)	0.74(0.44-1.24)	1.08(0.64-1.81)	0.96	
Tumor location							0.16
Distal	411(42.6)	Ref	1.04(0.67-1.62)	0.92(0.59-1.44)	1.12(0.72-1.74)	0.86	
Proximal	553(57.4)	Ref	0.70(0.49-1.00)	0.71(0.50-1.02)	0.73(0.50-1.05)	0.06	
BMI in FFQ1							0.26
<Median	402(49.9)	Ref	0.74(0.47-1.17)	0.83(0.52-1.31)	1.08(0.69-1.68)	0.97	
≥Median	403(50.1)	Ref	0.89(0.58-1.36)	0.69(0.44-1.08)	0.76(0.48-1.20)	0.11	
Physical activity in FFQ1							0.70
<Median	402(49.9)	Ref	0.72(0.47-1.11)	0.65(0.42-1.03)	0.84(0.54-1.30)	0.20	
≥Median	403(50.1)	Ref	0.83(0.53-1.30)	0.79(0.50-1.24)	0.89(0.56-1.39)	0.46	
Western dietary pattern in FFQ1							0.06
<Median	402(49.9)	Ref	0.59(0.36-0.95)	0.89(0.57-1.38)	1.09(0.70-1.68)	0.68	
≥Median	403(50.1)	Ref	0.75(0.49-1.15)	0.68(0.43-1.05)	0.64(0.40-1.01)	0.03	
Prudent dietary pattern in FFQ1							0.97
<Median	402(49.9)	Ref	0.81(0.53-1.24)	0.74(0.48-1.15)	0.89(0.57-1.37)	0.39	
≥Median	403(50.1)	Ref	0.62(0.38-0.99)	0.85(0.54-1.33)	0.74(0.46-1.16)	0.26	

Abbreviations: 5-FU = 5-fluorouracil; LV = leucovorin; IFL = irinotecan, 5-fluorouracil, leucovorin; FFQ = food frequency questionnaire; BMI = body mass index

[a] Hazards ratios and *P*-values based on subgroup-specific quartiles and quartiles medians.

[b] Multivariable-adjusted model adjusted for age (continuous), sex (male, female), treatment arm, T-stage (T1-2, T3-4), number of positive nodes (1-3, 4+), performance status (ECOG0, ECOG 1-2), tumor location (proximal, distal, or missing), clinical bowel obstruction or perforation (yes, no), race (White, Black, other), valid FFQ1 (yes, no), consistent aspirin use (yes, no), insurance status (private/Self-pay, Medicare/Medicaid/military/other/none), time-varying energy intake,

BMI, physical activity, Western dietary pattern, prudent dietary pattern (all time-varying variables are continuous).
[c] Interaction term built as a cross-product of median household income and the covariate of interest as binary variables.
[d] Baseline performance status: Performance status 0 = fully active; Performance status 1 = restricted in physically strenuous activity but ambulatory and able to carry out light work; Performance status 2 = ambulatory and capable of all self-care but unable to carry out any work activities, up and about more than 50% of waking hour.

Supplementary Table 4. Joint impact of race and income on cancer recurrence and mortality.[a]

	White & High Income	White & Low Income	Black & High Income	Black & Low Income
Household income, Median(Q1-Q3)	51824 (45138-62330)	34489 (30905-37526)	44375 (43890-52056)	31032 (25384-35632)
# Event/At Risk	#200/452	#159/394	#5/17	#28/67
DFS HR	Ref	0.85 (0.69 - 1.05)	0.66 (0.27 - 1.62)	0.80 (0.53 - 1.20)
P-value		0.13	0.36	0.29
# Event/At Risk	#172/452	#131/394	#5/17	#22/67
RFS HR	Ref	0.82 (0.65 - 1.04)	0.67 (0.27 - 1.66)	0.74 (0.47 - 1.16)
P-value		0.10	0.39	0.19
# Event/At Risk	#159/452	#135/394	#5/17	#25/67
OS HR	Ref	0.90 (0.71 - 1.14)	1.00 (0.41 - 2.48)	0.89 (0.58 - 1.38)
P-value		0.38	>0.99	0.61

[a] Multivariable-adjusted model adjusted for age (continuous), sex (male, female), treatment arm, T-stage (T1-2, T3-4), number of positive nodes (1-3, 4+), performance status (ECOG 0, ECOG 1-2), tumor location (proximal, distal, or missing), clinical bowel obstruction or perforation (yes, no), valid FFQ1 (yes, no), consistent aspirin use (yes, no), insurance status (private/self-pay, Medicare/Medicaid/military/other/none), time-varying energy intake, BMI, physical activity, Western dietary pattern, prudent dietary pattern (all time-varying variables are continuous).

Supplementary Figure 1. Derivation of the study cohort.

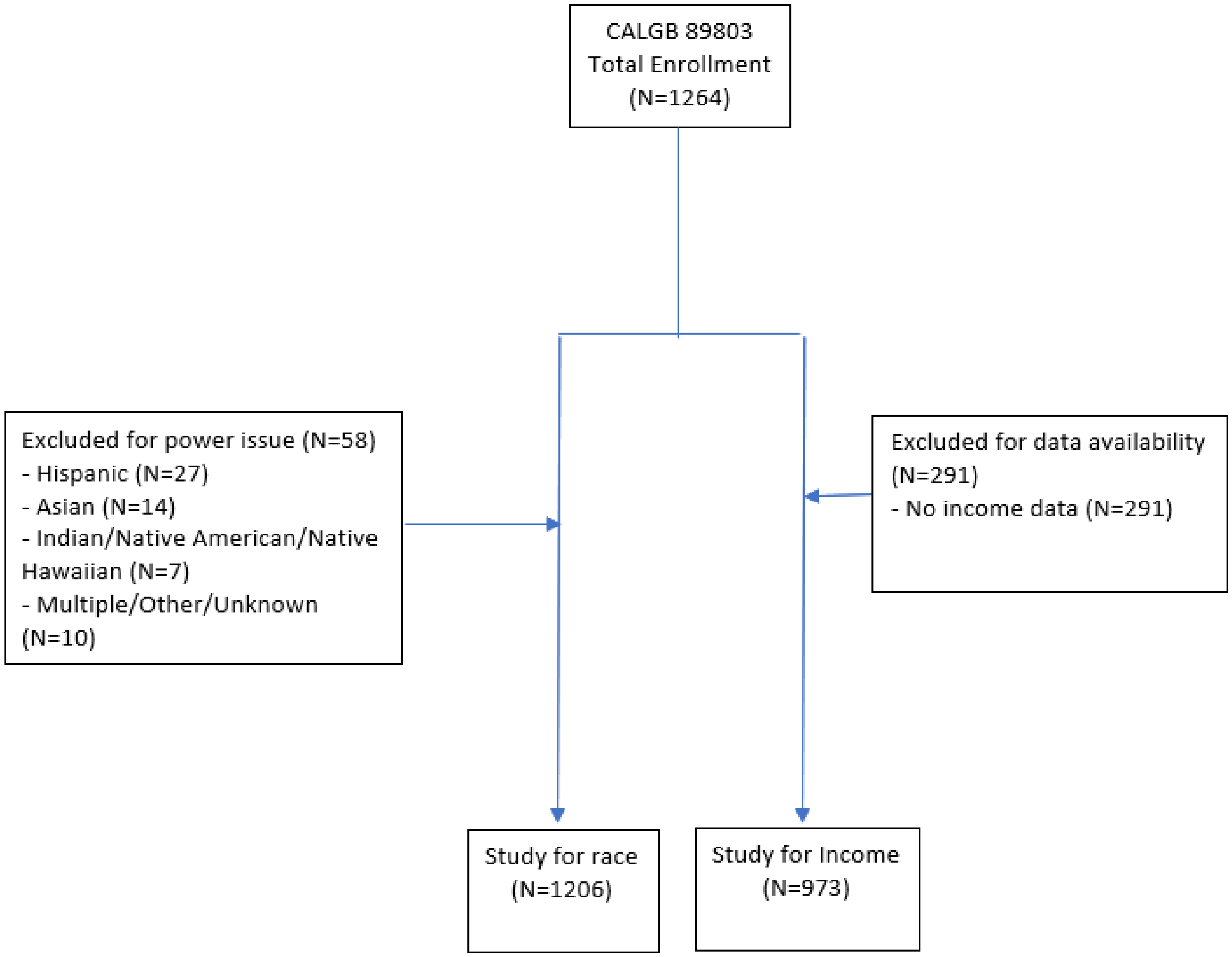

Supplementary Figure 2. Forest plot of race, colon cancer recurrence, and mortality.

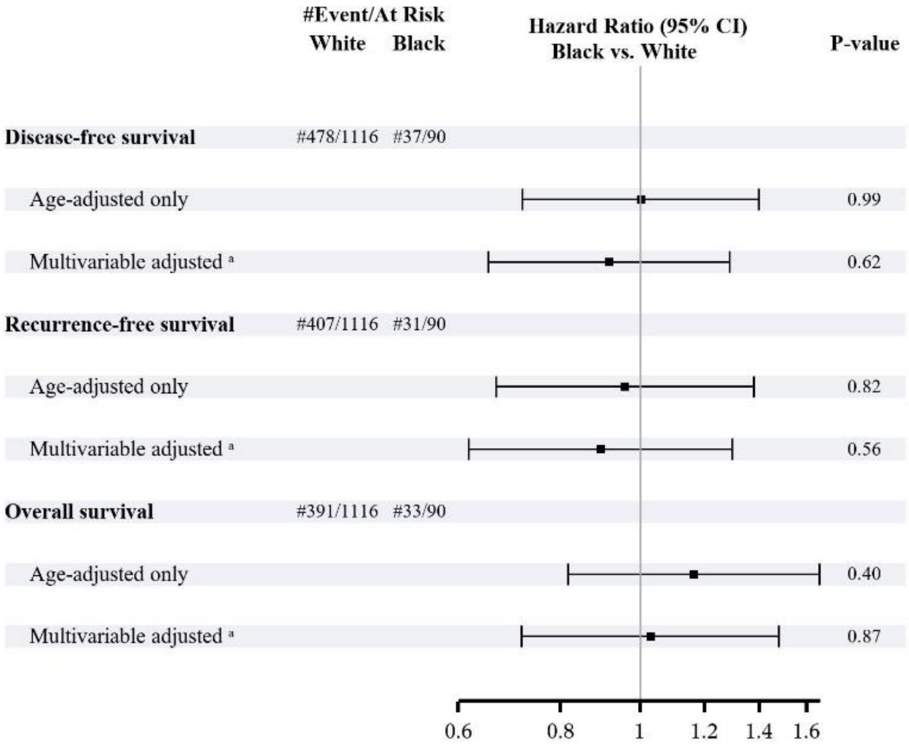

Supplementary Figure 3. Forest plot of income quartile, colon cancer recurrence, and mortality.

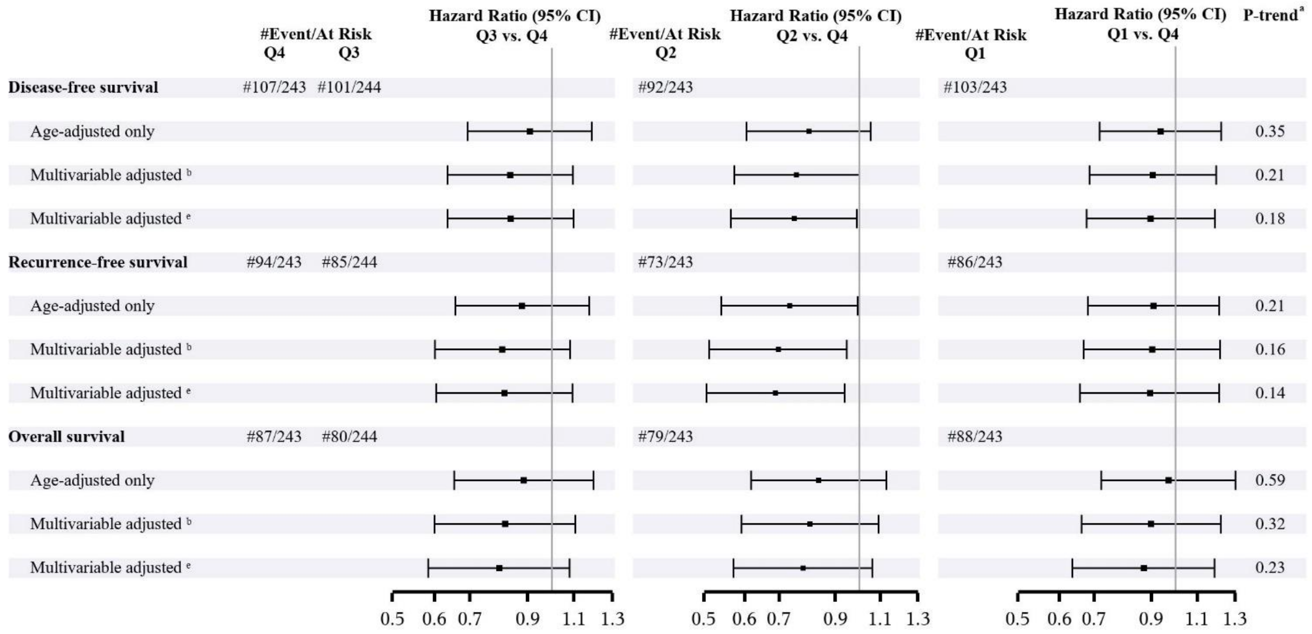

Supplement: pkab034_Supplementary_Data [file pkab034_supplementary_data.pdf]
